# Time is now to consider how we evaluate person-centred care—the role of patient-reported outcomes

**DOI:** 10.3389/frhs.2025.1578037

**Published:** 2025-06-13

**Authors:** Claudia Rutherford, Jan Boehnke, Joanne Greenhalgh, Vaibhav Tyagi, Tanya McCance, Brendan McCormack

**Affiliations:** ^1^ Faculty of Medicine and Health, The University of Sydney Susan Wakil School of Nursing and Midwifery, The University of Sydney, Sydney, NSW, Australia; ^2^ Faculty of Medicine and Health, Sydney Quality of Life Office, The University of Sydney, Sydney, NSW, Australia; ^3^ School of Health Sciences, University of Dundee, Dundee, United Kingdom; ^4^ School of Sociology and Social Policy, University of Leeds, Leeds, United Kingdom; ^5^ Institute of Nursing and Health Research, Ulster University, Belfast, United Kingdom

**Keywords:** person-centred care (PCC), patient-reported outcomes, evaluation, measurement framework, research

## Abstract

Person-centred care refers to health care that is respectful of and responsive to personal experiences, preferences, needs, goals and values of service users. Despite the growing recognition of the value of patient-reported outcome measures, they are rarely used as evaluation endpoints in person-centred care research and care practices. This paper contributes to knowledge by examining the opportunities and challenges of using patient-reported outcome measures to measure person-centred care. Our focus is not the collection and feedback of patient-reported outcomes to enact person-centred care. We discuss differences between patient- and person-reported outcomes and their role in assessing person-centred care. We also challenge some existing measurement practices and usage of existing patient-reported outcome measures. We critically discuss some potential consequences of current practices, and present possible solutions. We do not have all the answers, and we urge those working in the field of patient-reported measurement to collectively come together to find solutions. With this perspective article, we aim to start the conversation to think differently about how we evaluate person-centred care and propose areas of enquiry that incorporate patient-reported outcomes into the evaluation of person-centred care.

## Introduction

This paper aims to further our understanding of patient-reported measurement practices and improve *how* we evaluate person-centred care. *What* should be measured in this space has been previously reported (1). This paper is structured in four parts. First, we provide definitions for the key concepts covered in this paper: person-centred care, person-centred practice, and patient-reported outcomes. Second, we consider the role of patient-reported outcomes as evaluation endpoints in person-centred care research and care practices. Our focus is on patient-reported outcomes measuring the outcomes of person-centred care, not the collection and feedback of patient-reported outcomes to enact person-centred care, on which much has been published (2–4). Third, we critically reflect on measurement practices and usage of patient-reported outcome measures in person-centred care research. Finally, we end with a discussion of some potential consequences of current measurement practices and possible solutions for how the field might consider the inclusion of patient-reported outcomes in evaluative models of person-centred care. Our paper contributes to knowledge by setting out the opportunities and challenges of using patient-reported outcome measures to measure person-centred care.

### Key concepts and the need for clarity

Healthcare and healthcare practice is dominated by complex language and person-centred healthcare and assessing its outcomes is no different. Like Alice in Wonderland, sometimes it seems that a word “can mean just what we choose it to mean”, rather than there being an explicit and consistent use of words in this field. With that challenge in focus, we offer our perspective on essential key terms.

### Treating a patient as a whole person

The concept of treating a patient as a whole person and standards for person-centred caring were proposed back in 1981, with the development of a measure that enabled evaluation of the concept of treating a patient as a whole person, the Standards for Person-Centred Caring (SPCC) (5). The SPCC focused on assessing person-centred, rather than disease-centred issues through measurable structure, process and outcome criteria. Since then, several frameworks and standards of person-centred care (6, 7) and person-centred practice, as well as measures (or questionnaires) to assess them, have been developed and used (8). We have seen exponential growth of research assessing both patient and healthcare provider, particularly nurses, perceptions of person-centred caring and practices (9). More recently, the concept of *person-centredness* has emerged in healthcare guidance and policy, stressing approaches that focus on healthcare relationships and interactions that consider the whole life of a/the person.

### Person-centred care and person-centred practice

Person-centred care refers to healthcare that is respectful of, and responsive to, the preferences, needs, goals and values of service users. It is “*a way of practising or engaging with service users that is focused on their beliefs and values…their wants, needs, hopes and dreams—in deciding on care, and deciding on how best to deliver care. It's a relationship-based, partnership model where the person is at the centre of the decision-making, and the elements of the system fit around that, rather than the other way around,*” (10). It therefore requires a whole-systems understanding of, and commitment to, person-centredness as a philosophy for how care is organised, provided, and subsequently evaluated.

An increasing body of research has found person-centred care associated with many positive outcomes. For example, patients reported improved physical function, emotional state and quality of life; staff reported improvements in satisfaction and consultation time (11, 12); and supporting integrated care at the service level (13).

**Figure 1:**
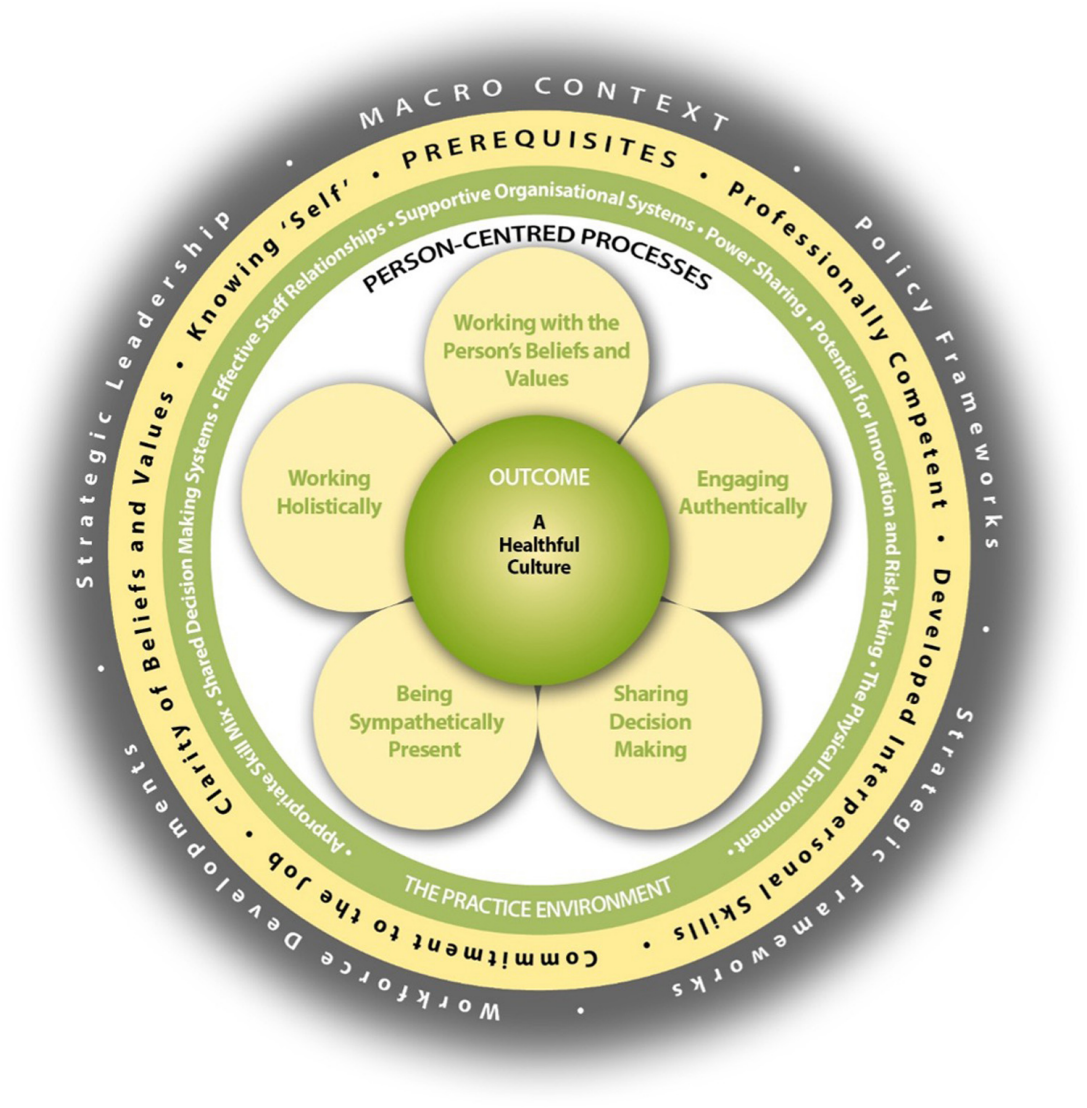
The person-centred practice framework (7).

Person-centred practice on the other hand, embraces the core philosophy of person-centred care, but contends that providing such care is unsustainable without applying the same values and principles to care providers. One framework that makes person-centred practice explicit and operationalises it as a whole-systems philosophy for the purpose of application and subsequent evaluation is the Person-Centred Practice Framework (7). Figure 1 depicts the relationship between the five domains of the Person-centred Practice Framework. The first domain, *prerequisites*, focuses on the attributes of staff. The second, the *practice environment*, focuses on the context in which healthcare is experienced. The third, the *person-centred processes*, focuses on ways of engaging that are necessary to create connections between persons. The fourth, the *outcome*, which is the result of effective person-centred practice. These four domains are set within the fifth domain, *the macro context* which reflects factors (regionally within country, nationally, internationally and globally) that are strategic and political in nature that influence the development of person-centred practices (7). To reach the centre of the framework, the attributes of staff must first be considered, as a prerequisite to managing the practice environment, to engage effectively through person-centred processes. This ordering ultimately leads to the achievement of the *outcome*, the central component of the framework, described as a healthful workplace culture, and with all of this influenced and shaped by the macro context. This ordering and layering is important as it highlights the impact of context (workplace culture) on the ability of individual clinicians to operationalise their qualities as person-centred practitioners, i.e., without a conducive context, sustaining effective person-centred practice cannot be realised. It is also important to recognise that there are relationships and overlap between the constructs within each domain, again showing the need for a whole-systems understanding of person-centred healthcare that ensures an organisation-wide responsibility for quality of care and not just individual clinician responsibility.

The Person-Centred Practice Framework has evolved over two decades of research and development activity and offers a common language and shared understanding of person-centred practice (14). In a broader sense, the Framework provides a quality assessment/assurance evaluation framework consisting of structure, process and outcome quality indicators. Use and adoption of this framework in research, practice, education and policy is widespread and the past two decades have seen a growth in research that focuses on evaluating the processes and outcomes arising from the implementation of person-centred care (14). Person-centred care is a dynamic multidisciplinary field in its own right, with an international community dedicated to innovating and improving healthcare (15). In this paper, we consider whether patient-reported outcomes fit within the fourth domain and central component of the Person-Centred Practice Framework and examine the extent to which they have been applied in a way that captures the process and impacts of a person-centred philosophy.

### Patient-reported outcomes

A patient-reported outcome (PRO) is “*a measurement based on a report that comes directly from the patient (i.e., study subject) about the status of a patient's health condition without amendment or interpretation of the patient's response by a clinician or anyone else*.” (16) They encompass a variety of measurable outcomes of care from the patient's perspective, including disease symptoms, side-effects of treatment, functioning, and health-related quality of life (HRQL) (17, 18). Over the past two decades, the added value of PRO data has been recognised and increasingly included as important endpoints in clinical research and to support labelling claims in drug development (16), and there is growing support from governments and professional organisations for using patient-reported outcome measures (PROMs) in healthcare to support person-centred care (19–22). They have also been used to judge the degree to which a hospital provides good quality care or improvements in patient-clinician communication—assessed through both PROMs and patient-reported experience measures (PREMs) that assess the impact of the process of care on the patient's experience. For example, patients’ perceptions of the structure and processes of care delivery (e.g., patient satisfaction), experience with healthcare services and care providers (e.g., patient-provider communication, care coordination), and patient activation (e.g., shared decision-making, self-efficacy/autonomy) (23). PREMs are classified as functional or relational. Functional PREMs examine practical issues (e.g., availability of facilities) while relational PREMs examine the patients’ experience of their relationships during treatment (e.g., did they feel listened to) (23). Several PREMs are available to assess person-centred care (8). Healthcare providers use both PROMs and PREMs in various ways to improve different aspects of patient care (24).

## The role of patient-reported outcomes as evaluation endpoints in person-centred care

Many of the outcomes (measured by PROMs) and *experiences* are useful measures for assessing person-centred care and can be linked to constructs included in the Person-Centred Practice Framework. However, we question, as McClimans does so eloquently, “how can measurement, which relies on standardisation, represent patients perspectives, which, if not idiosyncratic, are at least variable and changeable?” (25) And how do we factor into our measurement individual health-related preferences, needs, goals and values when in research we require rigorous standardised measurement tools to enable between-group and within-group comparisons; that is, assessing with the same questions, response options and scoring methods in all participants at each assessment time-point?

HRQL and symptom burden are PROs commonly used in comparative effectiveness research and health service evaluation as they are outcomes considered important to patients and useful for clinical decision-making. However, they are often aggregated without accounting for differences between individual beliefs, values, wants, needs and goals for healthcare, which are fundamental to person-centred care. Aggregating data is useful in healthcare if we want to evaluate the effectiveness of a particular treatment or our overall health service or practices. However, this aggregation is less important for individual patient care, and on its own, arguably not conducive to person-centred care. Two people with the same disease and treatment could have the same improvement in, for example, HRQL outcomes, but what we don't know, or rarely assess, is whether those improvements were meaningful to, or desired by, the individual person (26–28). Further, existing PROMs are often developed to allow for between-individual comparisons (nomothetic approach) and therefore include a standard set of pre-selected items which are presented to all participants. Albeit selected through a rigorous process and retained as the best set of items representing the outcomes selected as important to patients to measure, their completion does not allow for individual preferences, needs, goals and values for those PROs to be captured. This raises the question about whether PROs provide a mechanism for capturing and evaluating person-centred care?

Within healthcare, the term *outcome* refers to end-results or consequences of treatment, interventions, or healthcare (29). The PROMs used reflect indicators within healthcare that are based on *quality* of outcomes and impacts of health conditions and interventions from the perspective of the person experiencing them. Here, quality has to do with a person's perceived state and value of something, particularly their life or part of that life (in the form of outcomes of a healthcare procedure). However, the aspects of life that a person *values* are limited to the aspects represented in the PROM(s) used. This standardised measurement through use of existing PROMs implies that everyone values the same aspects represented and only captures what is represented. If a key element of person-centred care is the tailoring of care to address individual beliefs, values, wants, needs and goals for healthcare, then how can we reflect that potential heterogeneity of these when PROMs are standardised? Perhaps what is at the heart of the problem is the tension between the need to demonstrate effectiveness via standardised measurement with the principles of person-centred care, which is inherently individualised and tailored.

The concept of personhood lies at the heart of person-centredness in all its guises. Whilst a review of concepts and philosophies of personhood is beyond the scope of this paper, we draw on previous work to articulate personhood through modes of “being” (being in place, in relationship, with self, in social context and in time) (30, 31). In actively being, we draw on all kinds of knowledge and life experience to shape the way we exist in these modes. We are also in a constant process of change that is neither static nor fixed. A PRO is a static or fixed outcome whereas personhood is constantly changing and transforming so that the outcome measured is only a moment in time. Outcomes are of value, but only in any given time and in the specific context in which they were assessed. A shift in approaches to measurement in this context represents a shift in focus from “what is the matter with you” to “what matters to you” (32). This is something that has been embraced as a healthcare movement, but to which little systematic outcome measurement has been applied (33, 34). We need person-reported measurement that considers an individual persons’ preferences, needs, goals and values, and then a way of standardising that evaluation for the purpose of rigorous measurement.

## Critical reflection on measurement challenges and use of patient-reported outcome measures in person-centred care research

### Complexity of person-centred care

Despite the recognised value of person-centred care and of the persons’ perspectives on healthcare, measuring whether person-centred care has occurred, and if it has occurred, its impact on patient outcomes presents ongoing challenges to researchers, clinicians, and patients (35). Whilst the complexity of person-centred healthcare as a whole-systems approach to practice presents one set of unique evaluation challenges, another part of the measurement problem is how we define, operationalise, and evaluate person-centred care. Whilst a definition of person-centred practice is offered in the Person-Centred Practice Framework, within that there is no clear articulation of what *person*-related outcomes should be evaluated to assess whether person-centred care has actually been provided and led to improved patient outcomes.

### Lack of consistency in the person-centred care discourse

Another problem is the interchangeable use of person- and patient-centred care despite published differences (36) and no published distinction between patient- vs. person-reported outcomes (measures) or between patient- vs. person-centred outcomes (measures). Whilst we have agreement about what constitutes a PRO, to the best of our knowledge, we lack a published or internationally accepted definition of person-reported outcomes for the purpose of measurement. Person-reported outcome has been used in published literature, however, the articles reported on what we know as *PRO*s, using these terms interchangeably (e.g., person-reported outcomes of health status but no definitions; others provided the same definition for person-reported outcomes as the FDA definition for PROs) (37, 38). One group described the term *person* being relevant when referring to proxy-reports given by relatives, caregivers or other health professionals when the patient was unable to report on their health, or when outcomes related to general populations, for example, when developing preference-based measures (39). Interchangeable use makes it difficult to tease out distinct differences between the concepts and how people are working within them. Further, it precludes agreement about models that operationalise person-centred care and person-reported outcomes for the purpose of measurement.

### Limitations of existing evaluation practices

Despite the lack of clarity, established programs of work aim to measure person-centred care. However, evaluation outcomes have included a narrow range such as the quality of the care given, assessed using different patient-reported measures that collect varying information, rather than broader processes and practices within a whole system approach (8). A 2014 review of commonly used approaches and tools to measure person-centred care found a large number of tools available, without agreement about which to use to measure person-centred care, with no one questionnaire covering all aspects of person-centred care (8). Further, no single valid and reliable measurement tool has been recommended for general use (40). Poorly described definitions of constructs measured and lack of conceptual frameworks that underpinned the measurement models may be a large part of the problem. Capturing the complexity of person-centred care and the influencing individual, contextual and cultural factors should be considered in measurement frameworks.

Since the 2014 review, important contributions to developing evaluation models of person-centred care and practices of measuring and improving person-centred care are being made. A new instrument underpinned by the Person-Centred Practice Framework has been developed—the Person-Centred Practice Inventory (PCPI). This is available in both staff and patient versions and enables assessment of how person-centred practice is perceived (41). The PCPI evaluates the process and experience of person-centred practice and care, but not outcomes in this context; perhaps a gap that PROs could somewhat fill. Additional work by McCance et al. has developed and tested eight person-centred key performance indicators for evaluating and improving person-centred nursing practice (42, 43). However, how these process measures align with outcome measures remains a challenge. Santana et al. (2018) developed a conceptual model of person-centred care consisting of structure, process and outcome components that includes PROs as one of two outcome domains (44). Importantly, the value of PROs is recognised and recommended as an evaluative outcome of the impact of person-centred care. This model has informed several quality improvement initiatives. For example, work from Canada developed a core group of person-centred quality indicators applicable across healthcare sectors and contexts that provides standardised metrics to measure person-centred care to help drive the changes needed to improve the quality of healthcare that is person-centred. These quality indicators can be used by healthcare systems to monitor and evaluate the delivery of person-centred care, identify the gaps, and make the changes needed to improve the quality of care (45). However, only one PRO, general health, is an included quality indicator.

### Uncertainty about what to measure in person-centred care

As highlighted earlier, a key measurement problem is lack of agreement about what should be measured—is it the enactment of person-centred care (i.e., as a process) or the anticipated outcomes of person-centred care—but what are these and how do we decide? Without answering these questions we cannot determine whether we have adequate PRO(M)s for the purpose of evaluating person-centred care and it may in part be the reason for the lack of practical examples of how PROs can be useful in person-centred care. The challenge we face is often construed as us needing to develop methods to measure a PRO at the individual level that considers individual preferences, needs, goals and values for treatment and outcomes, but which can still be aggregated despite such variability to demonstrate effective person-centred care based on between-individual measurements. Nevertheless, it is well-understood that measures of PROs are always only validated for specific purposes (46–48), that they depend on the epistemic goals and positions of developers and users (25, 49, 50), and finally, international initiatives such as the development of core outcome sets and similar assessment frameworks recognise that usually more than one outcome is required (51). Describing the goal of the process as finding a single measure to represent the multidimensional concept of person-centred care may be posing the wrong question and setting the endeavour up for failure.

## Potential consequences of current measurement practices and possible solutions

PROs such as symptom control and maintaining or improving HRQL are important outcomes of person-centred care but only tell us part of the person-centred story. Constraining measurement to disease burden, as is with the HRQL approach to measuring function and health status, moves us away from considering how a person perceives and reacts to their health status. But we know that one's HRQL perception is influenced by an interaction of personal and environmental influences that determine quality of life (52). Using only standardised measures of HRQL or health status would not enable consideration of individual goals, needs, and preferences for the quality of individual life and would fail to comprehensively assess the different components of person-centred care practices. Both aspects are needed to collectively reflect evidence of successful person-centred care. This is where PREMs may be beneficial to capture certain aspects such as whether a personalized care plan was developed or whether patients felt involved in decision-making. These experiences are shared across individuals, even if the care plans themselves differ. Such questions operate on a meta-level: the content of the care plan may vary, but the existence and co-creation of that plan are measurable and comparable.

Pairing PREMs with PROMs allows for meaningful analysis of patient experiences of care and services. This notion is reflected in several international initiatives. The Organisation for Economic Co-operation and Development (OECD) set a new international standard for patient-reported outcomes and experiences through its Patient-Reported Indicator Surveys (PaRIS) initiative, where countries worked together to develop, standardise and implement a new generation of indicators that measure the outcomes and experiences of healthcare that matter most to people (53). The International Consortium for Health Outcomes Measurement (ICHOM) developed several standard sets of outcomes based on patient priorities (54). The sets of outcomes mostly focus on patient-centred outcomes, but some do include experience of care measures (55). The World Health Organization (WHO) also recognizes the importance of PROs and experiences, emphasizing their role in patient safety and quality of care (56), and promoting their use to improve healthcare quality and outcomes (57). These initiatives emphasize people-centredness, a concept that underpins frameworks like the WHO's People-Centred Health Care Framework (58) and the OECD's People-Centred Health Systems framework (59).

However, we contend that this only tells part of the story of person-centred care and its outcomes. The term *patient* does not encompass the whole person in the context of healthcare (60) and reduces an individual person to their disease and treatment. Person-centredness is fundamentally about individual goals, considering the social context and the kind of life that a person wants to live. So, to capture these dynamic caring practices we need to move beyond measurement of patient-reported indicators of clinical effectiveness towards more holistic measures that evaluate PROs in the context of the whole person including individual goals and preferences for treatment, personal values, and social and cultural contexts. But if we advocate for respecting the whole person then we need to operate within a social model of health. Social models of health recognize that our health is influenced by a wide range of individual, interpersonal, organizational, social, environmental, political and economic factors (61).

Our measurement frameworks should be reconsidered in light of how person-reported outcomes/experiences fits within the context of person-centredness. But the challenge is how to capture these subtleties in our patient-reported measures. “*No two people are the same”* is at the core of person-centredness so one might argue that we cannot aggregate outcomes data for everyone. One might further argue that we cannot standardise these outcomes because everyone is different so then what do we measure to capture the essence of person-centred care? Improved person-centredness is an implied driver of quality of life assessments in clinical practice (62). However, this approach does not address the challenge of how to capture person-centred care within a much broader understanding of a person's life experiences, values, beliefs and preferences, before, during and after care giving, the environmental context, the interactions between care providers and service users, and the perceptions of the care providers. Aggregate data allows us to evaluate whether we are doing/achieving person-centred care and whether that care is improving patient outcomes, whatever they might be for the individual. But to achieve this we need to individualise our care and therefore our assessments. So, then how do we evaluate person-centred care and marry aggregate and individual data? This is the real challenge.

Perhaps as a first step, our person-centred care measurement models and measures should factor in PROs and working with the person's beliefs and values within broader life domains and social contexts. Additionally, we need agreement about what we believe the outcomes of person-centred care will be. In person-centred care evaluation, perhaps we should be asking patients what they hope to achieve with their treatment, rather than confining evaluation of person-centred care to preselected standardised outcomes.

Several approaches may provide some solutions for our measurement conundrum. In the needs-based approach, rather than asking directly about a function, it is possible to inquire about the needs that could be satisfied by that function (63). The Needs-Based approach to quality of life is based on the individual's possibility of fulfilling their expectations and needs in life (64). Similarly, the underlying propositions of the Schedule for the Evaluation of Individual Quality of Life are that quality of life is individual in nature and that an individual's judgment of their overall quality of life is constructed from their assessment of their level of functioning/satisfaction in discrete domains of life which they consider to be important (65). Goal Attainment Scaling is a measurement tool that allows patients to set individual goals, together with their treating healthcare professional (66, 67); and individual-generated indices allow patients to develop their own assessment content (e.g., most concerning or impacting symptoms) (68–70). Despite being developed over 30 years ago, these approaches have not been widely adopted. Reasons for this are unclear but may be in part due to the contradiction we highlight in this paper, i.e., the acknowledgement of the uniqueness of individual experience of healthcare matched by the need for universality for resource planning and decision-making. Most PROMs have been developed to offer an option for between-individual comparisons. This leads to instruments where the same set of symptoms, health or quality of life impacts are presented to all individuals. And while these are at least today usually the result of a robust multi-round, mixed methods, and stakeholder-informed process, there is no guarantee that the content represents the needs, goals, values and preferences for treatment for every individual. Individualised measures might allow for individual goals/needs for treatment to be captured. But given that the content of such measures is then individualised and heterogeneous, it is unclear whether patient-responses to such assessments can be aggregated for between-individual comparisons, and what such aggregates would mean.

Finally, predictive models or computerized adaptive testing (CAT) may offer increases in efficiency identifying relevant subsets of questions selected from the full patient-reported questionnaires, triggered by and optimising measurement precision for what patients say is important to them based on their preferences, needs, goals and values. CATs are a method between fully standardised (such as questionnaires) and fully individualised assessments (such as individual-generated indices or goal attainment scaling). A computer program is used to select questions from a larger pool to tailor the assessment to the individual without loss of scale precision or content validity if items being selected measure the same construct (71). They have been used in this way to assess PROs in health-related research for the past two decades (72).

We would argue that while healthcare cultures are shifting their focus to improvement, approaches to measurement continue to privilege standardised, quantifiable data and information that can be used for standardisation (73). Despite over 30 years of developments in patient-centred and then person-centred care, quantitative measurement continues to dominate, despite doing little to inform stakeholders about the person-centredness of a health system. So, understanding whether a person recovers is of course a good and important thing, and we do not want to move away from assessing whether a patient improved, recovered, lived well and so on. However, understanding the extent of the healthcare experience and recovery in terms of what it means to an individual and one's ability to engage in the five modes of being is what is needed to shift from measuring outcomes of health status to measuring person-centred care. Further, we need a shift in perception that addressing a patients HRQL is not within the remit of healthcare providers or that they lack time to do it (74).

When we talk about PROs, we are essentially talking about an outcome that we want to assess or measure. In person-centred care, we need common agreement and understanding about what the *outcome(s)* is that we are interested in. We also need theoretical models that operationalise these *outcomes* of interest and how we can measure and assess whether we are truly delivering person-centred care and working within person-centred caring practices. The literature often reverts to proxy measures in terms of outcomes for person-centred care; a reflection of difficulty in trying to define what we mean in this space. But in order to demonstrate the value of person-centred cultures to healthcare organisations and the significance of person-centred outcomes for patients, families, carers and staff, we need greater clarity in our definitions, concepts and models, and to embrace theory-driven evaluation designs that fully embrace mixed-methodologies and capture the diversity of experiences among all stakeholders, as well as demonstrating effectiveness (73). Needing standardised aggregated assessment for evaluative research designs should not be an excuse for assessing the *wrong* things or omitting other important aspects that are perhaps more challenging to measure and interpret.

## Data Availability

The original contributions presented in the study are included in the article/Supplementary Material, further inquiries can be directed to the corresponding authors.
